# Assessing the potential of deep learning for protein-ligand docking

**Published:** 2025-08-12

**Authors:** Alex Morehead, Nabin Giri, Jian Liu, Pawan Neupane, Jianlin Cheng

**Affiliations:** 1 NERSC, Lawrence Berkeley National Laboratory, Berkeley, California, USA.; 2 Electrical Engineering & Computer Science, NextGen Precision Health, University of Missouri, Columbia, Missouri, USA.

**Keywords:** Deep learning, Molecular docking, Protein-ligand interactions, Benchmarks

## Abstract

The effects of ligand binding on protein structures and their *in vivo* functions carry numerous implications for modern biomedical research and biotechnology development efforts such as drug discovery. Although several deep learning (DL) methods and benchmarks designed for protein-ligand docking have recently been introduced, to date no prior works have systematically studied the behavior of the latest docking and structure prediction methods within the *broadly applicable* context of (1) using predicted (apo) protein structures for docking (e.g., for applicability to new proteins); (2) binding multiple (cofactor) ligands concurrently to a given target protein (e.g., for enzyme design); and (3) having no prior knowledge of binding pockets (e.g., for generalization to unknown pockets). To enable a deeper understanding of docking methods’ real-world utility, we introduce PoseBench, the first comprehensive benchmark for *broadly applicable* protein-ligand docking. PoseBench enables researchers to rigorously and systematically evaluate DL methods for apo-to-holo protein-ligand docking and protein-ligand structure prediction using *both* primary ligand and multi-ligand benchmark datasets, the latter of which we introduce for the first time to the DL community. Empirically, using PoseBench, we find that (1) DL co-folding methods generally outperform comparable conventional and DL docking baseline algorithms, yet popular methods such as AlphaFold 3 are still challenged by prediction targets with novel protein-ligand binding poses; (2) certain DL co-folding methods are highly sensitive to their input multiple sequence alignments, while others are not; and (3) DL methods struggle to strike a balance between structural accuracy and chemical specificity when predicting novel or multi-ligand protein targets. Code, data, tutorials, and benchmark results are available at https://github.com/BioinfoMachineLearning/PoseBench.

## 1 Introduction

The field of drug discovery has long been challenged with a critical task: determining the structure of ligand molecules in complex with proteins and other key biomolecules [1]. As accurately identifying such complex structures (in particular multi-ligand structures) can yield advanced insights into the binding dynamics and functional characteristics (and thereby, the medicinal potential) of numerous protein complexes *in vivo*, in recent years, significant resources have been spent developing new experimental and computational techniques for protein-ligand structure determination [2]. Over the last decade, machine learning (ML) methods for structure prediction have become indispensable components of modern structure determination at scale, with AlphaFold 2 for protein structure prediction being a hallmark example [3, 4].

As the field has gradually begun to investigate whether proteins in complex with other types of molecules can faithfully be modeled with ML (and particularly deep learning (DL)) techniques [5–7], several new works in this direction have suggested the promising potential of such approaches to protein-ligand structure determination [8–11]. Nonetheless, it remains to be shown the extent to which the latest of such (docking and co-folding-based) DL methods can adequately generalize to the context of binding novel or uncommon protein-ligand interaction (PLI) pockets and multiple interacting ligand molecules (e.g., which can alter the chemical functions of various enzymes) as well as whether such methods can faithfully model amino acid-specific types of PLIs natively found in crystallized biomolecular structures.

To bridge this knowledge gap, our contributions in this work are as follows:

- We introduce the first unified benchmark for protein-ligand docking and structure prediction that evaluates the performance of several recent DL-based baseline methods (DiffDock-L, DynamicBind, NeuralPLexer, RoseTTAFold-All-Atom, Chai-1, Boltz-1, and AlphaFold 3) as well as conventional algorithms (P2Rank + AutoDock Vina) for primary and *multi*-ligand docking, which suggests that DL co-folding methods generally outperform conventional algorithms yet remain challenged by novel or uncommon prediction targets.
- In contrast to several recent works using crystal protein structures for protein-ligand docking [12, 13], the docking benchmark results we present in this work are all within the context of *standardized* input multiple sequence alignments (MSAs) and high accuracy *apo-like* (i.e., AlphaFold 3-predicted) protein structures (see Appendix D) without specifying known binding pockets, which notably enhances the broad applicability of this study’s findings.
- Our newly proposed benchmark, PoseBench, enables specific insights into necessary areas of future work for accurate and generalizable biomolecular structure prediction, including that DL methods struggle to balance faithful modeling of native PLI fingerprints (PLIFs) with structural accuracy during pose prediction and that some DL co-folding methods (AlphaFold 3) are more dependent than others (Boltz-1, Chai-1) on the availability of input MSAs.
- Our benchmark results also highlight the importance of including challenging (out-of-distribution) datasets when evaluating future DL methods while measuring their ability to recapitulate amino acid-specific PLIFs with an appropriate new metric that we introduce in this work.

## 2 Results and discussion

**Fig. 1:**
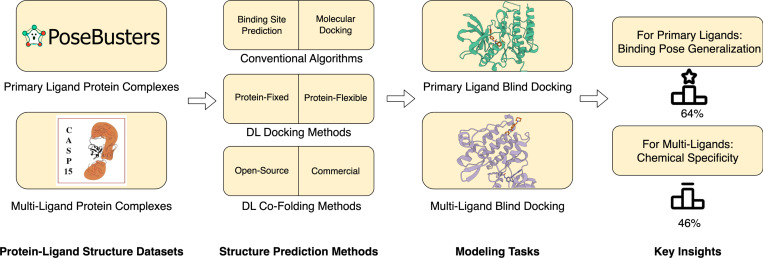
Overview of PoseBench, our comprehensive benchmark for *broadly applicable* DL modeling of primary and multi-ligand protein complex structures. Baseline algorithms within the benchmark include a range of the latest DL docking and co-folding methods, both open-source and commercially restrictive, as well as conventional algorithms for docking. Key observations derived using PoseBench include the discontinuity between structure and interaction modeling performance for novel or uncommon prediction targets and the heavy reliance of key DL co-folding methods on MSA-based input features to achieve high structural accuracy.

In this section, we present PoseBench’s results for primary and multi-ligand protein-ligand docking and structure prediction and discuss their implications for future work, as succinctly illustrated in Figure 1. Note that across all experiments, for generative methods, we report their performance metrics in terms of the mean and standard deviation across *three* independent runs of each method to gain insights into their inter-run stability and consistency. Key metrics include a method’s percentage of structurally accurate ligand pose predictions with a (heavy atom centroid) root mean square deviation (RMSD) less than 2 (1) Å (i.e., (c)RMSD *≤* 2 (1) Å); its percentage of structurally accurate pose predictions that are also chemically valid according to the PoseBusters software suite (i.e., RMSD *≤* 2 Å & PB-Valid), which can be affected by the post-hoc application of structural relaxation driven by computationally expensive molecular dynamics (MD) simulations [14] (i.e., with relaxation); and our newly proposed Wasserstein matching score of its amino acid-specific predicted PLIFs (PLIF-WM). We formally define these metrics in Section 4.4. For interested readers, in Appendix C, we report the average runtime and memory usage of each baseline method to determine which methods are the most efficient for real-world structure-based applications, and in Appendix G we present supplementary results.

### 2.1 Astex Diverse results

**Fig. 2:**
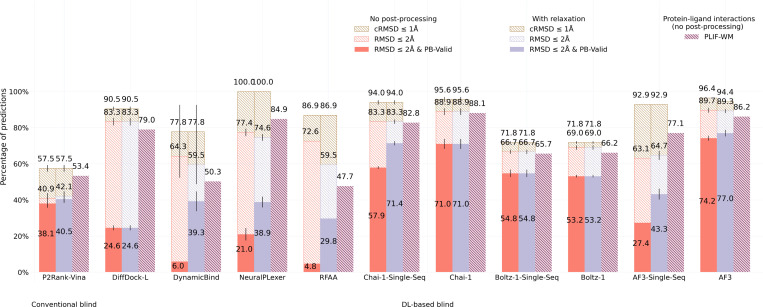
Astex Diverse primary ligand docking success rates (n=85).

Containing PLI structures deposited in the RCSB Protein Data Bank (PDB) [15] up until 2007, most of the well-known Astex Diverse dataset’s structures [16] are present in the training data of each baseline method, yet benchmarking results for this dataset (n=85), shown in Figure 2, indicate that only DL co-folding methods achieve higher structural and chemical accuracy rates (RMSD *≤* 2 Å & PB-Valid) than the conventional docking baseline AutoDock Vina combined with P2Rank for PLI binding site prediction to facilitate blind molecular docking. Interestingly, nearly all baseline methods identify the correct PLI binding pocket approximately 90% of the time, yet only the DL co-folding methods Chai-1 [17], Boltz-1 [18], and AlphaFold 3 (AF3) [11] achieve a reasonable balance between their rates of structural and chemical accuracy and chemical specificity (PLIF-WM), with the single-sequence (i.e., MSA-ablated) version of AF3 being a notable exception. These results suggest that DL co-folding methods have learned the most comprehensive representations of this dataset’s input sequences, yet only the DL co-folding method Chai-1 maintains strong performance without the availability of diverse input MSAs. One likely explanation for this phenomenon is that Chai-1’s training relied upon the availability of amino acid sequence embeddings generated by the protein language model ESM2 [19] in addition to features derived from input MSAs, which may have imbued the model with rich MSA-*independent* representations for biomolecular structure prediction.

### 2.2 DockGen-E results

**Fig. 3:**
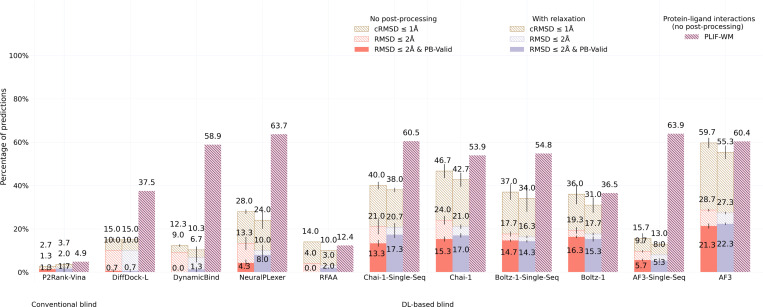
DockGen-E primary ligand docking success rates (n=122).

As visualized in Figure 3, results with our new DockGen-E dataset of biologically relevant PLI complexes deposited in the PDB up to 2019 (n=122) demonstrate that only the latest DL co-folding methods can locate a sizable fraction of structurally accurate PLI binding poses represented in this dataset. As such methods may have previously seen these PLI structures in their respective training data, it is surprising that even the latest AF3 model fails to identify a structurally and chemically accurate pose for more than 75% of the dataset’s complexes. Further, for Chai-1, Boltz-1, and AF3, their single-sequence variants achieve higher chemical specificity than their MSA-based versions, which may indicate that for these methods MSA features obfuscate primary sequence knowledge in favor of evolution-averaged (i.e., amino acid-generic) representations. The overall lower range of PLIF-WM values achieved by each method for this dataset further suggests the increased chemical modeling difficulty of this dataset’s complexes compared to those presented by the Astex Diverse dataset. A potential source of these difficulties is that each of this dataset’s complexes represents a functionally distinct PLI binding pocket (as codified by ECOD domains [20], see [21] for more details) compared to data deposited in the PDB before 2019. As such, it is likely that Chai-1, Boltz-1, and AF3 are “overfitted” to the most common types of PLI structures in the PDB and may overlook several uncommon types of PLI binding pockets present in nature.

### 2.3 PoseBusters Benchmark results

**Fig. 4:**
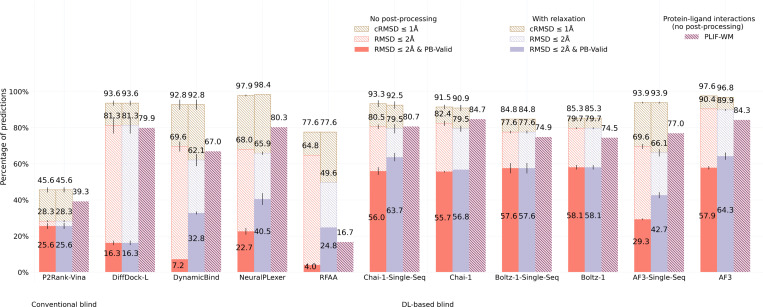
PoseBusters Benchmark primary ligand docking success rates (n=130/308).

With approximately half of its PLI structures deposited in the PDB after AF3 and Boltz-1’s maximum-possible training data cutoff of September 30, 2021 (n=308 total, filtered to n=130 for subsequent analyses), the PoseBusters Benchmark dataset’s results, presented in Figure 4, indicate once again that DL co-folding methods achieve top performance compared to conventional and DL docking baseline methods. Nonetheless, we observe an interesting phenomenon whereby Chai-1 strikes a balance of structural and chemical accuracy and chemical specificity comparable to that of the best-performing AF3, even without input MSAs, potentially suggesting that Chai-1 achieves stronger binding pose generalization for this dataset than AF3. Moreover, with the single-sequence version of AF3, we again observe significant degradations in its overall performance, whereas running Chai-1 with input MSAs achieves higher chemical specificity at the cost of marginal structural accuracy compared to running it in single-sequence mode. These observations highlight the importance in future work of carefully studying why and how the *training* of biomolecular structure generative models can be influenced to varying degrees by the availability and composition of diverse input MSAs.

### 2.4 CASP15 results

**Fig. 5:**
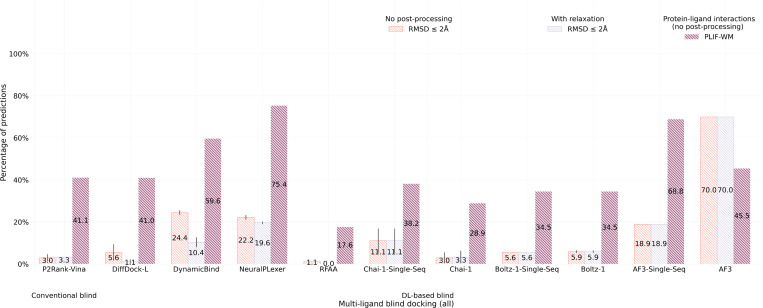
CASP15 multi-ligand docking success rates (n=13).

**Fig. 6:**
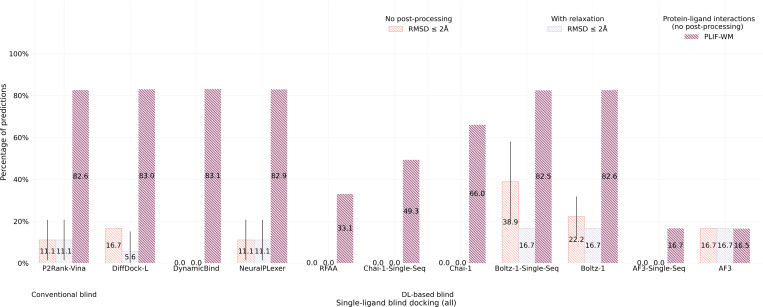
Extended Data Figure 1 — CASP15 single-ligand docking success rates (n=6).

As a new dataset of novel and challenging PLI complexes on which no method has been trained, the CASP15 dataset’s multi-ligand results (n=13), illustrated in Figure 5, indicate that most methods fail to adequately generalize to multi-ligand prediction targets, yet AF3 stands out in this regard (only) when provided input MSAs. As many of these CASP15 multi-ligand targets represent large, highly symmetric protein complexes, it is likely that additional evolutionary information in the form of MSAs has improved AF3’s ability to predict higher-order protein-protein interactions for these targets, yet interestingly its improved rate of structural accuracy comes at the cost of its protein-*ligand* chemical specificity (in comparison to its single-sequence results). For the CASP15 dataset’s single-ligand (i.e., primary ligand) results (n=6) presented in Figure 6, this trend is subverted in that conventional docking and open-source DL co-folding methods such as AutoDock Vina, NeuralPLexer, and Boltz-1 outperform all other recent DL co-folding methods in modeling crystalized PLIFs while achieving comparable rates of structural accuracy. Given the small size of the CASP15 dataset, it is reasonable to conclude that DL methods, in particular some of the latest co-folding methods, *may* be challenged to predict protein-ligand complexes containing novel PLIs. In the following Section 2.5, we will explore this latter point in greater detail by analyzing the protein-ligand binding similarities between common PDB training data and this benchmark’s evaluation datasets.

### 2.5 Exploratory analyses of results

In this section, we explore a range of questions to study the common “failure” modes of the baseline methods included in this work, to outline new directions for future research and development efforts in drug discovery.

#### Research Question 1:

What are the most common types of protein-ligand complexes that *all* baseline methods fail to predict?

**Fig. 7:**
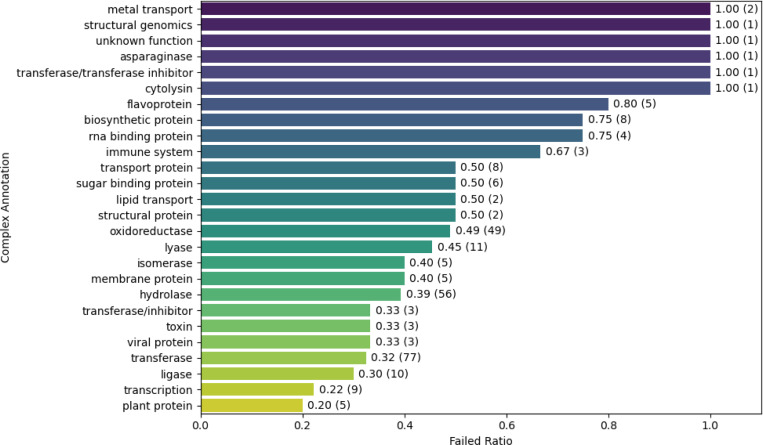
Extended Data Figure 2 — Function annotations of the PLI complexes all methods mispredicted (n=129).

*→* To address this query, we first collect all ligand pose predictions that no method could predict with structural and chemical accuracy (according to the metric RMSD ≤ 2 Å & PB-Valid). For each of these “failed” ligand poses, we retrieve the PDB’s functional annotation of the protein in complex with this ligand and construct a histogram to visualize the frequency of these (failed complex) annotations. The results of this analysis are presented in Figure 7, in which we see that metal transport proteins, flavoproteins, biosynthetic proteins, RNA binding proteins, immune system proteins, and oxidoreductases are commonly mispredicted by all baseline methods such as Chai-1 and RoseTTAFold-All-Atom (RFAA) [7], suggesting these classes of proteins may be largely unaddressed by the most recent DL methods for PLI structure prediction. To illuminate potential future research directions, in the next analysis, we investigate whether this pattern persists specifically for AF3, the most accurate DL *co-folding method according to our benchmarking results*.

#### Research Question 2:

What are the most common types of protein-ligand complexes that highly-accurate DL *co-folding* methods such as AF3 fail to predict?

**Fig. 8:**
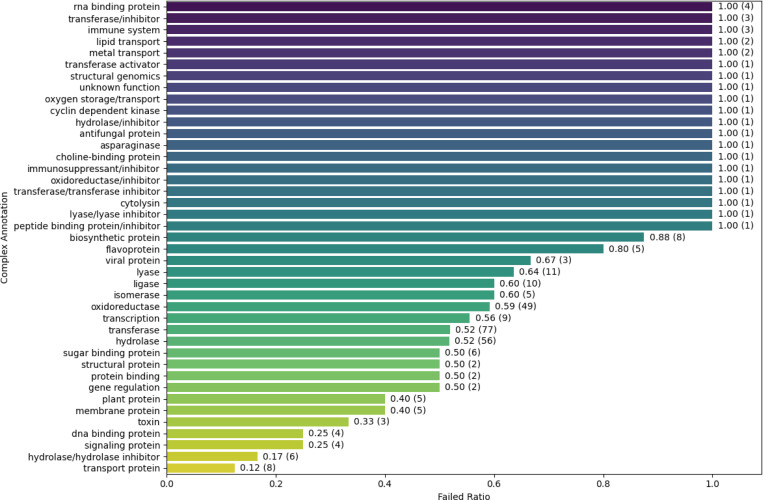
Extended Data Figure 3 — Function annotations of the PLI complexes AF3 mispredicted (n=171).

*→* For this follow-up question, we link all of AF3’s failed ligand predictions with corresponding protein function annotations available in the PDB to understand which types of PLI complexes AF3 finds the most difficult to predict (to understand its predictive coverage of important molecular functions). Similar to the answer to our first research question, Figure 8 shows that, in order of difficulty, AF3 is most challenged to produce ligand poses of high structural and chemical accuracy for ligand-bound RNA binding proteins, immune system proteins, metal transport proteins, biosynthetic proteins, flavoproteins, lyases, and oxidoreductases. As several of these classes of proteins have not been well represented in the PDB over the last 50 years (e.g., immune system and biosynthetic proteins), in future work, it will be important to ensure that either the performance of new DL methods for PLI structure prediction is expanded to support accurate modeling of these uncommon types of ligand-bound proteins or a broadly applicable fine-tuning method for uncommon types of interactions is proposed.

#### Research Question 3:

Is *lack* of protein-ligand binding pose homology to PDB training data (inversely) correlated with the prediction accuracy of each method?

**Fig. 9:**
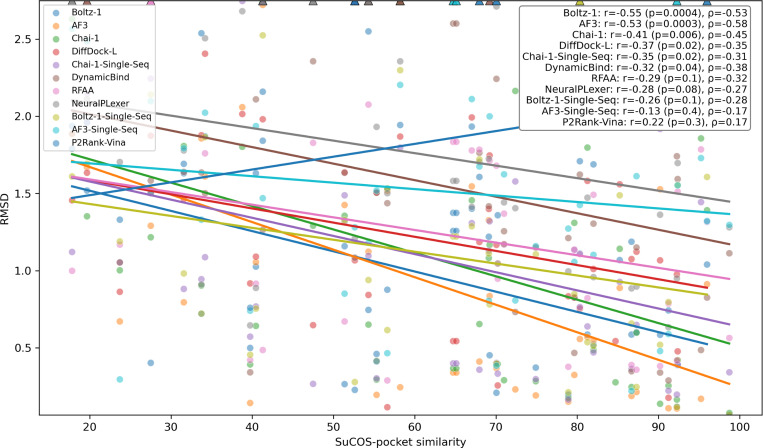
Extended Data Figure 4 — Relationship between methods’ PoseBusters Benchmark prediction accuracy (RMSD) and each complex’s training set similarity (SuCOS-pocket similarity) (n=41).

**Fig. 10:**
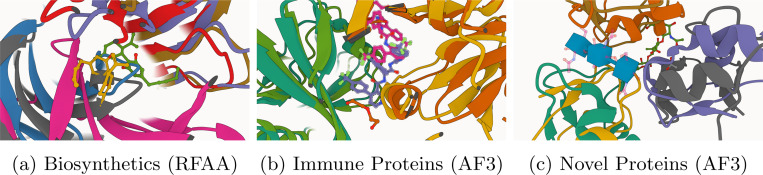
Extended Data Figure 5 — Examples of baseline methods’ three failure modes discussed in this work.

*→* To understand the impact of protein-ligand binding pose similarity on the performance of each baseline method, we use the PLINDER data resource [22] to identify (n=41/130) cluster representatives of the PoseBusters Benchmark dataset based on the product of each complex’s ligand (structure and feature-based) Combined Overlap Score (SuCOS) [23] and its protein binding pocket’s (structure and sequence-based) similarity [24], as none of this subset’s prediction targets are contained in any method’s training dataset. For these cluster representatives, we then calculate the Pearson and Spearman correlations (and p-values) between each method’s complex prediction accuracy (i.e., ligand pose RMSD) and the complex’s maximum SuCOS-binding pocket-based similarity to any complex deposited in the PDB before AF3’s training dataset cutoff of September 30, 2021. Figure 9 reveals that *all* DL methods’ performance is correlated with a complex’s similarity to common PDB training data, with (MSA-based) Boltz-1, AF3, and Chai-1 exhibiting the strongest and most statistically significant (*p <* 0.05) correlations. Like concurrent work assessing the performance of co-folding methods in novel prediction settings [24], our findings suggest that although the current generation of deep learning models (*both* docking and co-folding methods) for protein-ligand docking and structure prediction can occasionally make accurate predictions for truly novel (SuCOS-pocket similarity *<* 30) protein-ligand complexes, such methods rely (at least in large part) on *recapitulating* protein-ligand binding patterns seen during training to make accurate predictions for unseen complexes (n.b., while interestingly for conventional docking methods such as P2Rank-Vina, this trend is not observed). We conclude our quantitative analyses with an illustration of the different failure modes of each baseline method, as depicted in Figure 10. In this figure, we illustrate that DL methods such as RFAA and AF3 commonly struggle to accurately predict the structure of ligand-binding biosynthetic and immune system proteins, suggesting that these (uncommon) types of PLIs are not well addressed by the current generation of DL-based structure prediction methods, suggesting future research opportunities for interaction-specific modeling (e.g., through the use of fine-tuning or preference optimization).

## 3 Conclusions

In this work, we have introduced PoseBench, a unified, broadly applicable benchmark and toolkit for studying the performance of methods for protein-ligand docking and structure prediction. Benchmarking results with PoseBench, summarized in Table 1, suggest that DL co-folding methods generally outperform conventional and DL docking baselines yet remain challenged to predict complexes containing novel protein-ligand binding poses, with AF3 performing best overall when deep MSAs are available for a target protein (Chai-1 otherwise), regardless of the availability of homologous proteins. Further, we find that several DL methods face difficulties balancing the structural accuracy of their predicted poses with the chemical specificity of their induced protein-ligand interactions, highlighting that future methods may benefit from the introduction of *physico-chemical* loss functions or sampling techniques to bridge this performance gap. Lastly, we observe that some (but not all) DL co-folding methods are highly dependent on the availability of diverse input MSAs to achieve high structural prediction accuracy (e.g., AF3 but not Chai-1 or Boltz-1), underscoring the need in future work to elucidate the impact of the availability of MSAs and protein language model embeddings on the training dynamics of biomolecular structure prediction methods. As a publicly available resource, PoseBench is flexible to accommodate new datasets, methods, and analyses for protein-ligand docking and structure prediction.

## 4 Methods

### 4.1 PoseBench

The overall goal of PoseBench, our newly designed benchmark for protein-ligand docking and structure prediction, is to provide the research community with a centralized resource with which one can systematically measure, in a variety of macromolecular contexts, the methodological advancements of new conventional and DL methods proposed for this domain. In the following sections, we describe PoseBench’s design and composition (as portrayed in Figure 1) and how we have used PoseBench to evaluate several recent DL docking and co-folding methods (as well as a strong conventional baseline algorithm) for protein-ligand structure modeling.

### 4.2 Benchmark datasets

As shown in Table 1, PoseBench provides users with *broadly applicable*, preprocessed versions of four datasets with which to evaluate existing or new protein-ligand structure prediction methods: Astex Diverse [16], PoseBusters Benchmark [12], and the new DockGen-E and CASP15 PLI datasets that we have manually curated in this work.

#### Astex Diverse dataset.

The Astex Diverse dataset is a collection of 85 PLI complexes composed of various drug-like molecules and cofactors known to be of pharmaceutical or agrochemical interest, where a primary (representative) ligand is annotated for each complex. This dataset can be considered an easy benchmarking dataset for methods trained on recent data contained in the PDB in that most of its complexes (deposited in the PDB up to 2007) are known to overlap with the commonly used PDBBind 2020 (time-split) training dataset [25, 26] containing complexes deposited in the PDB before 2019. As such, including this dataset for benchmarking allows one to estimate the *breadth* of a method’s structure prediction capabilities for important primary ligand protein complexes represented in the PDB.

To perform unbound (apo) protein-ligand docking with this dataset, we used AF3 to predict the structure of each of its protein complexes, with all ligands and cofactors excluded. We then optimally aligned these predicted protein structures to the corresponding crystal (holo) PLI complex structures using a PLI binding site-focused structural alignment performed using PyMOL [27], where each binding site is defined as all amino acid residues containing crystallized heavy atoms that are within 10 Å of any crystallized ligand heavy atom. To enable the broad availability of PoseBench’s benchmark datasets in both commercial and academic settings, we also provide unbound (apo) protein structures predicted using the MIT-licensed ESMFold model [19], although in Section 2 we report results using AF3’s predicted structures as the default data source. We further note that on average across all benchmark datasets and methods, AF3’s predicted structures improve baseline docking methods’ structural accuracy rates by 5–10%.

#### PoseBusters Benchmark dataset.

Version 2 of the the popular PoseBusters Benchmark dataset [12], which we adopt in this work, contains 308 recent primary ligand protein complexes deposited in the PDB from 2019 onwards. Accordingly, in contrast to Astex Diverse, this dataset can be considered a moderately difficult benchmark dataset for baseline methods, since many of its complexes do not directly overlap with the most commonly used PDB-based training data. Important to note is that, among all baseline methods, AF3 and Boltz-1 used the most recent PDB training data cutoff of September 30, 2021, which motivated us to report the results in Section 2.3 for only the subset of PoseBusters Benchmark complexes (n=130) deposited in the PDB after this date. Like Astex Diverse, for the PoseBusters Benchmark dataset, we used AF3 (and ESMFold) to predict the *apo* protein structures of each of its complexes and then performed our PyMOL-based structural binding site alignments.

#### DockGen-E dataset.

The original DockGen dataset [13] contains 189 diverse primary ligand protein complexes, each representing a functionally distinct type of PLI binding pocket according to ECOD domain partitioning [20, 21]. Consequently, this dataset can be considered PoseBench’s most difficult primary ligand dataset to model since its PLI binding sites are distinctly uncommon compared to those frequently found in the training datasets of all baseline methods, though it is important to note that these original DockGen complexes were deposited in the PDB from 2019 onward, making this benchmarking dataset partially overlap with the training datasets of baseline DL co-folding methods such as Chai-1, Boltz-1, and AF3. Nonetheless, in line with our initial hypotheses, the benchmarking results in Section 2 demonstrate that no baseline method can adequately predict the PLI binding sites and ligand poses represented by this bespoke subset of the PDB, suggesting that *all* baseline DL methods have yet to learn *broadly applicable* representations of protein-ligand binding.

Unfortunately, the original DockGen dataset contains only the primary protein chains representing each novel binding pocket after filtering out all non-interacting chains and cofactors in a given biological assembly (bioassembly), which considerably *reduces* the biophysical context provided to baseline methods to make reasonable predictions. As such, we argue for the need to construct a new dataset that challenges baseline methods (in particular DL co-folding methods) to predict full bioassemblies containing novel PLI binding pockets, which we address with our enhanced version of DockGen called DockGen-E.

To construct DockGen-E, we collected the original DockGen dataset’s PLI binding pocket annotations for each complex. We then retrieved the corresponding first bioassembly listed in the PDB to obtain each PDB entry’s biologically *relevant* complex, filtering out DockGen complexes for which the first bioassembly could not be mapped to its original PLI binding pocket annotation (which indicates these original DockGen PLI binding pockets were initially not derived from the PDB’s corresponding first bioassembly). This procedure left 122 biologically relevant assemblies remaining for benchmarking. Like Astex Diverse and PoseBusters Benchmark, for DockGen-E, we then used AF3 (and ESMFold) to predict the unbound (apo) protein structures of each complex in the dataset and structurally aligned the predicted protein structures to their corresponding crystallized PLI binding sites using PyMOL.

#### CASP15 dataset.

To assess the *multi*-primary ligand (i.e., multi-ligand) modeling capabilities of recent methods for protein-ligand docking and structure prediction, with PoseBench, we introduce a preprocessed, DL-ready version of the CASP15 PLI dataset debuted as a first-of-its-kind prediction category in the 15th Critical Assessment of Techniques for Structure Prediction (CASP) competition held in 2022 [28]. The CASP15 PLI dataset is originally comprised of 23 protein-ligand complexes released in the PDB from 2022 onward, where we subsequently filter out 4 complexes based on (1) whether the CASP organizers ultimately assessed predictions for the complex and (2) whether they are nucleic acid-ligand complexes with no interacting protein chains. The 19 remaining PLI complexes, which contain a total of 102 (fragment) ligands, consist of a variety of ligand types including single-atom (metal) ions and large drug-sized molecules with up to 92 atoms in each (fragment) ligand. As such, this dataset is appropriate for assessing how well structure prediction methods can model interactions between different (fragment) ligands in the same complex, which can yield insights into the inter-ligand steric clash rates of each method. As with all other benchmark datasets, we used AF3 (and ESMFold) to predict the unbound (apo) structure of each protein complex in the dataset and then performed a PyMOL-based structural alignment of the corresponding PLI binding sites.

#### PLI similarity analysis between datasets.

For an investigation of the similarity of PLIs represented in each dataset, in Appendix E, we analyze the different types and frequencies of common, ProLIF-annotated protein-ligand binding pocket interactions [29] natively found within the common PDBBind 2020 training dataset and the Astex Diverse, PoseBusters Benchmark, DockGen-E, and CASP15 datasets, respectively, to quantify the diversity of the (predicted) interactions each dataset can be used to evaluate. In short, we find that the DockGen-E and CASP15 benchmark datasets are the *most dissimilar* compared to the common PDBBind 2020 training dataset, further illustrating the unique PLI modeling challenges offered by these evaluation datasets.

### 4.3 Formulated tasks

In this work, we developed PoseBench to focus our analysis on the behavior of different conventional and DL methods for protein-ligand structure prediction in a variety of macromolecular contexts (e.g., with or without inorganic cofactors present). With this goal in mind, below we formalize the structure prediction tasks currently available with PoseBench, with its source code flexibly designed to accommodate new tasks in future work.

#### Primary ligand blind docking.

For primary ligand blind docking, each baseline method is provided with a complex’s (multi-chain) protein sequence and an optional predicted (apo) protein structure as input along with its corresponding (fragment) ligand SMILES strings, where fragment ligands include the *primary* binding ligand to be scored as well as all cofactors present in the corresponding crystal structure. In particular, no knowledge of the complex’s PLI binding pocket is provided to evaluate how well each method can (1) identify the correct PLI binding pockets and (2) correct ligand poses within each pocket (3) with high chemical validity and (4) specificity for the pockets’ amino acid residues. After all fragment ligands are predicted, PoseBench extracts each method’s prediction of the primary binding ligand and reports evaluation results for these primary predictions.

#### Multi-ligand blind docking.

For multi-ligand blind docking, each baseline method is provided with a complex’s (multi-chain) protein sequence and an optional predicted (apo) protein structure as input along with its corresponding (fragment) ligand SMILES strings. As in primary ligand blind docking, no knowledge of the PLI binding pockets is provided, which offers the opportunity to evaluate not only PLI binding pocket and conformation prediction accuracy but, in the context of multi-binding ligands, also inter-ligand steric clash rates.

### 4.4 Metrics

#### Traditional metrics.

For PoseBench, we reference two key metrics in the field of structural bioinformatics: the root-mean-square deviation (RMSD) and local Distance Difference Test (lDDT) [30]. The RMSD between a predicted 3D conformation (with atomic positions ${\hat{x}}_{i}$ for each of the molecule’s n heavy atoms) and the ground-truth (crystal structure) conformation $\left(x_{i}\right)$ is defined as:

(1) $$\text{RMSD}=\sqrt{\frac{1}{n}\sum_{i=1}^{n}{\left\|{\hat{x}}_{i}-x_{i}\right\|}^{2}}.$$

The lDDT score, which is commonly used to compare predicted and ground-truth protein 3D structures, is defined as:

(2) $$\mathrm{lDDT}=\frac{1}{N}\sum_{i=1}^{N}\frac{1}{4}\sum_{k=1}^{4}\left(\frac{1}{\left|N_{i}\right|}\sum_{j\in N_{i}}\text{Θ}\left(\left|{\hat{d}}_{ij}-d_{ij}\right|<{\text{Δ}}_{k}\right)\right),$$

where N is the total number of heavy atoms in the ground-truth structure; $N_{i}$ is the set of neighboring atoms of atom i within the inclusion radius $R_{o}=15Å$ in the ground-truth structure, excluding atoms from the same residue; ${\hat{d}}_{ij}\left(d_{ij}\right)$ is the distance between atoms i and j in the predicted (ground-truth) structure; ${\text{Δ}}_{k}$ are the distance tolerance thresholds (i.e., 0.5Å 1Å 2Å and 4Å); Θ(x) is a step function that equals 1 if x is true, and 0 otherwise; and $\left|N_{i}\right|$ is the number of neighboring atoms for atom i. As originally proposed by Robin et al. [28], in this study, we adopt the PLI-specific variant of lDDT for scoring *multi*-ligand complexes, which calculates lDDT scores to compare predicted and ground-truth protein-(multi-)ligand complex structures following optimal (chain-wise and residue-wise) structural alignment of the predicted and ground-truth PLI binding pockets.

Lastly, we also measure the molecule validity rates of each predicted PLI complex pose using the PoseBusters software suite (i.e., PB-Valid) [12]. This suite runs several important chemical and structural sanity checks for each predicted pose including energy ratio inspection and geometric (e.g., flat aliphatic ring) assertions which provide a secondary filter of accurate poses that are also chemically and structurally meaningful.

#### New metrics.

The RMSD, lDDT, and PB-Valid metrics of a protein-ligand binding structure provide useful characterizations of how accurate and reasonable a predicted pose is. However, a key limitation of these metrics is that they do not measure how well a predicted pose resembles a native pose when comparing their induced PLIFs. Recently, [31] introduced a complementary benchmarking metric, PLIF-valid, assessing DL methods’ recovery rates of known PLIs. However, this metric only reports a strict recall rate of each method’s interaction types rather than a continuous measure of how well each method’s interactions match the *distribution* of crystalized PLIs. Moreover, in drug discovery, a primary concern when designing new drug candidates is ensuring they produce *amino acid-specific* types of interactions (and not others), hence we desire each baseline method to recall the correct types of PLIs for each pose and to avoid predicting (i.e., hallucinating) types of interactions that are not natively present. Consequently, we argue that an ideal PLI-aware benchmarking metric is a single continuous metric that assesses the recall and precision of a method’s predicted *distribution* of *amino acid-specific* PLIFs. To this end, we propose two new benchmarking metrics, PLIF-EMD and PLIF-WM.

For each PLI complex, PLIF-EMD measures the Earth mover’s distance (EMD) [32] between a method’s predicted histogram of PLI type counts u (specific to each type of interaction) and the corresponding native histogram v, where each histogram of interaction type counts is represented as a 1D discrete distribution. Formally, this equates to computing the Wasserstein distance between these two 1D distributions u and v as

(3) $$\text{PLIF-EMD}:=l_{1}(u,v)=\underset{\pi \text{∈Π}(u,v)}{\text{inf}}\int_{\text{ℝ}\times \text{ℝ}}|x-y|d\pi (x,y),$$

where Π(u,v) denotes the set of distributions on ℝ×ℝ whose marginals, u and v, are on the first and second factors, respectively. To penalize a baseline method for producing non-native interaction types, we unify the bins in each histogram before converting them into 1D discrete representations. Namely, to perform this calculation, each PLI is first represented as a fingerprint tuple of *<*ligand type, amino acid type, interaction type*>* as determined by the software tool ProLIF [29] and then grouped to count each type of tuple to form a histogram. As such, a lower PLIF-EMD value implies a better continuous agreement between predicted and native interaction histograms. PLIF-WM, derived from PLIF-EMD, assesses the Wasserstein matching (WM) score of a pair of PLIF histograms. Specifically, to obtain a more benchmarking-friendly score ranging from 0 to 1 (higher is better), we define PLIF-WM as

(4) $$\text{PLIF-WM}:=1-\frac{\text{PLIF-EMD}-{\text{PLIF-EMD}}_{\text{min}}}{{\text{PLIF-EMD}}_{\max}-{\text{PLIF-EMD}}_{\min}},$$

where ${\text{PLIF-EMD}}_{\min}$ and ${\text{PLIF-EMD}}_{\max}$ denote the minimum (best) and maximum (worst) values of PLIF-EMD, respectively. As a metric normalized relative to each collection of the latest baseline methods, PLIF-WM allows one to quickly identify which of the latest methods has the greatest capacity to produce realistic distributions of PLIs. As a practical note, we use SciPy 1.15.1 [33] to provide users of PoseBench with an optimized implementation of PLIF-EMD and thereby PLIF-WM.

### 4.5 Baseline methods and experimental setup

#### Overview.

We designed PoseBench to answer specific modeling questions for PLI complexes such as (1) which types of methods (if any) can predict both common and uncommon PLI complexes with high structural and chemical accuracy and (2) which most accurately predict multi-ligand structures without steric clashes? In the following sections, we discuss which types of methods we evaluate in our benchmark and how we evaluate each method’s predictions for PLI complex targets.

#### Method categories.

As illustrated in Figure 1, to explore a range of the most well-known or recent methods to date, we divide PoseBench’s baseline methods into one of three categories: (1) conventional algorithms, (2) DL docking algorithms, and (3) DL co-folding algorithms.

As a representative algorithm for conventional protein-ligand docking, we pair AutoDock Vina (v1.2.5) [34] for molecular docking with P2Rank for protein-ligand binding site prediction [35] to form a strong conventional (blind) docking baseline (P2Rank-Vina) for comparison with DL methods. To represent DL docking methods, we include DiffDock-L [13] for docking with static protein structures and DynamicBind [9] for flexible docking. Lastly, to represent some of the latest DL co-folding methods, we include NeuralPLexer [10], RFAA [7], Chai-1 [17], Boltz-1 [18] (versus Boltz-2 [36] for sake of time-split benchmarking validity), and AF3 [11]. For interested readers, each method’s input and output data formats are described in Appendix F.

#### Prediction and evaluation procedures.

The PLI complex structures each method predicts are subsequently evaluated using different structural and chemical accuracy and molecule validity metrics depending on whether the targets are primary or multi-ligand complexes. In Section 4.4, we provide formal definitions of PoseBench’s evaluation metrics. Note that if a method’s prediction raises any errors in subsequent scoring stages (e.g., due to missing entities or formatting violations), the prediction is excluded from the evaluation.

#### Primary ligand evaluation.

For primary ligand targets, we report each method’s percentage of (top-1) ligand conformations within 2 Å of the corresponding crystal ligand structure (RMSD *≤* 2 Å), using 1 Å to instead assess whether the predicted ligand’s heavy atom centroid (i.e., binding pocket) was correct (cRMSD *≤* 1 Å), as well as the percentage of such ”correct” ligand conformations that are also considered to be chemically and structurally valid according to the PoseBusters software suite [12] (RMSD *≤* 2 Å & PB-Valid). Importantly, as described in Section 4.4, we also report each method’s new PLIF-WM scores to study the relationship between its structural accuracy and chemical specificity.

#### Multi-ligand evaluation.

Similar to the protein-ligand scoring procedure employed in the CASP15 competition [28], for multi-ligand targets, we report each method’s (top-1) percentage of ”correct” (binding site-superimposed) ligand conformations (RMSD *≤* 2 Å) as well as violin plots of the RMSD and PLI-specific lDDT scores of its protein-ligand conformations across all (fragment) ligands within the benchmark’s multi-ligand complexes (see Appendix G for these plots). Notably, this latter metric, referred to as lDDT-PLI, allows one to evaluate specifically how well each method can model protein-ligand structural interfaces. Additionally, we report each method’s PB-Valid rates (calculated once for each multi-ligand complex) and PLIF-WM scores.

## 5 Related work

### Structure prediction of PLI complexes.

The field of DL-driven protein-ligand structure determination was largely sparked with the development of geometric deep learning methods such as EquiBind [26] and TANKBind [37] for direct (i.e., regression-based) prediction of bound ligand structures in protein complexes. Notably, these predictive methods could estimate localized ligand structures in complex with multiple protein chains as well as the associated complexes’ binding affinities. However, in addition to their limited predictive accuracy, they have more recently been found to frequently produce steric clashes between protein and ligand atoms, notably hindering their widespread adoption in modern drug discovery pipelines.

### Protein-ligand structure prediction and docking.

Shortly following the first wave of predictive methods for protein-ligand structure determination, DL methods such as DiffDock [8] demonstrated the utility of a new approach to this problem by reframing protein-ligand docking as a generative modeling task, whereby multiple ligand conformations can be generated for a particular protein target and rank-ordered using a predicted confidence score [38]. This approach has inspired many follow-up works offering alternative formulations of this generative approach to the problem [7, 9–11, 13, 17, 18, 36, 39–52], with some of such follow-up works also being capable of accurately modeling protein flexibility upon ligand binding or predicting binding affinities to a high degree of accuracy.

### Benchmarking efforts for protein-ligand complexes.

In response to the large number of new methods that have been developed for protein-ligand structure prediction, recent works have introduced several new datasets and metrics with which to evaluate newly developed methods [24], with some of such benchmarking efforts focusing on modeling single-ligand protein interactions [12, 22, 31, 53–56] and others specializing in the assessment of multi-ligand protein interactions [28]. One of the motivations for introducing PoseBench in this work is to bridge this gap by systematically assessing a selection of the latest (pocket-blind) structure prediction methods within both interaction regimes, using unbound (apo) protein structures with docking methods and challenging DL co-folding methods to predict full bioassemblies from primary sequences. Our benchmarking results in Section 2 demonstrate the relevance and utility of this comprehensive new evaluation suite for the future of protein-ligand modeling.

## Extended Data

## Supplementary Material

Supplement 1

## Figures and Tables

**Table 1:** PoseBench evaluation datasets of protein-(multi-)ligand structures.

	Astex Diverse	DockGen-E	PoseBusters Benchmark	CASP15
Ligand Type	Primary	Primary	Primary	Multi
Source	[16]		[12]	
Size (Total # Ligands)	85	122	130/308	102 (across 19 complexes) *→* 6 (13) single (multi)-ligand complexes
Training Data Homology	High	Moderate	Low	Low
Top-Ranked Method (w/ MSAs)	AF3	AF3	AF3	AF3
Top-Ranked Method (w/o MSAs)	Chai-1	Chai-1	Chai-1	NeuralPLexer

## Data Availability

The PoseBench datasets and benchmark results are available at https://zenodo.org/records/16791095 under a Creative Commons Attribution 4.0 International Public License, with further licensing discussed in Appendix A and detailed dataset documentation (e.g., of AF3’s predicted protein structure accuracy) provided in Appendix D.

## References

[1] Warren G.L., Do T.D., Kelley B.P., Nicholls A., Warren S.D.: Essential considerations for using protein–ligand structures in drug discovery. Drug Discovery Today 17(23–24), 1270–1281 (2012) 22728777 10.1016/j.drudis.2012.06.011

[2] Du X., Li Y., Xia Y.-L., Ai S.-M., Liang J., Sang P., Ji X.-L., Liu S.-Q.: Insights into protein–ligand interactions: mechanisms, models, and methods. International journal of molecular sciences 17(2), 144 (2016) 26821017 10.3390/ijms17020144 PMC4783878

[3] Jumper J., Evans R., Pritzel A., Green T., Figurnov M., Ronneberger O., Tunyasuvunakool K., Bates R., Žídek A., Potapenko A., : Highly accurate protein structure prediction with alphafold. Nature 596(7873), 583–589 (2021) 34265844 10.1038/s41586-021-03819-2 PMC8371605

[4] Abriata L.A.: The nobel prize in chemistry: past, present, and future of ai in biology. Communications Biology 7(1), 1409 (2024) 39472680 10.1038/s42003-024-07113-5 PMC11522274

[5] Dhakal A., McKay C., Tanner J.J., Cheng J.: Artificial intelligence in the prediction of protein–ligand interactions: recent advances and future directions. Briefings in Bioinformatics 23(1), 476 (2022) 10.1093/bib/bbab476 PMC8690157 34849575

[6] Harris C., Didi K., Jamasb A.R., Joshi C.K., Mathis S.V., Lio P., Blundell T.: Benchmarking generated poses: How rational is structure-based drug design with generative models? arXiv preprint arXiv:2308.07413 (2023)

[7] KrishnaR., WangJ., AhernW., SturmfelsP., VenkateshP., KalvetI., LeeG.R., Morey-BurrowsF.S., AnishchenkoI., HumphreysI.R., : Generalized biomolecular modeling and design with rosettafold all-atom. Science, 2528 (2024)10.1126/science.adl252838452047

[8] Corso G., Stärk H., Jing B., Barzilay R., Jaakkola T.: Diffdock: Diffusion steps, twists, and turns for molecular docking. arXiv preprint arXiv:2210.01776 (2022)

[9] Lu W., Zhang J., Huang W., Zhang Z., Jia X., Wang Z., Shi L., Li C., Wolynes P.G., Zheng S.: Dynamicbind: predicting ligand-specific protein-ligand complex structure with a deep equivariant generative model. Nature Communications 15(1), 1071 (2024) 10.1038/s41467-024-45461-2 PMC10844226 38316797

[10] Qiao Z., Nie W., Vahdat A., Miller T.F. III, Anandkumar A.: State-specific protein–ligand complex structure prediction with a multiscale deep generative model. Nature Machine Intelligence, 1–14 (2024)

[11] Abramson J., Adler J., Dunger J., Evans R., Green T., Pritzel A., Ronneberger O., Willmore L., Ballard A.J., Bambrick J., : Accurate structure prediction of biomolecular interactions with alphafold 3. Nature, 1–3 (2024) 10.1038/s41586-024-07487-w PMC11168924 38718835

[12] Buttenschoen M., Morris G.M., Deane C.M.: Posebusters: Ai-based docking methods fail to generate physically valid poses or generalise to novel sequences. Chemical Science (2024) 10.1039/d3sc04185a PMC10901501 38425520

[13] Corso G., Deng A., Fry B., Polizzi N., Barzilay R., Jaakkola T.: Deep confident steps to new pockets: Strategies for docking generalization. arXiv preprint arXiv:2402.18396 (2024)

[14] Eastman P., Pande V.: Openmm: A hardware-independent framework for molecular simulations. Computing in science & engineering 12(4), 34–39 (2010) 10.1109/MCSE.2010.27 PMC4486654 26146490

[15] Bank P.D.: Protein data bank. Nature New Biol 233(223), 10–1038 (1971)

[16] Hartshorn M.J., Verdonk M.L., Chessari G., Brewerton S.C., Mooij W.T., Mortenson P.N., Murray C.W.: Diverse, high-quality test set for the validation of protein-ligand docking performance. Journal of medicinal chemistry 50(4), 726–741 (2007) 17300160 10.1021/jm061277y

[17] Discovery C., Boitreaud J., Dent J., McPartlon M., Meier J., Reis V., Rogozhnikov A., Wu K.: Chai-1: Decoding the molecular interactions of life. bioRxiv, 2024–10 (2024)

[18] Wohlwend J., Corso G., Passaro S., Reveiz M., Leidal K., Swiderski W., Portnoi T., Chinn I., Silterra J., Jaakkola T., : Boltz-1 democratizing biomolecular interaction modeling. BioRxiv (2024)

[19] Lin Z., Akin H., Rao R., Hie B., Zhu Z., Lu W., Smetanin N., Verkuil R., Kabeli O., Shmueli Y., : Evolutionary-scale prediction of atomic-level protein structure with a language model. Science 379(6637), 1123–1130 (2023) 36927031 10.1126/science.ade2574

[20] Cheng H., Schaeffer R.D., Liao Y., Kinch L.N., Pei J., Shi S., Kim B.-H., Grishin N.V.: Ecod: an evolutionary classification of protein domains. PLoS computational biology 10(12), 1003926 (2014) 10.1371/journal.pcbi.1003926 PMC4256011 25474468

[21] Corso G., Deng A., Fry B., Polizzi N., Barzilay R., Jaakkola T.: The Discovery of Binding Modes Requires Rethinking Docking Generalization. International Conference on Learning Representations (ICLR) (2024). https://doi.org/10.5281/zenodo.10656052. 10.5281/zenodo.10656052

[22] Durairaj J., Adeshina Y., Cao Z., Zhang X., Oleinikovas V., Duignan T., McClure Z., Robin X., Kovtun D., Rossi E., : Plinder: The protein-ligand interactions dataset and evaluation resource. bioRxiv, 2024–07 (2024)

[23] Leung S., Bodkin M., Delft F., Brennan P., Morris G.: Sucos is better than rmsd for evaluating fragment elaboration and docking poses. ChemRxiv (2019)

[24] Škrinjar P., Eberhardt J., Durairaj J., Schwede T.: Have protein-ligand co-folding methods moved beyond memorisation? BioRxiv, 2025–02 (2025)

[25] Liu Z., Su M., Han L., Liu J., Yang Q., Li Y., Wang R.: Forging the basis for developing protein–ligand interaction scoring functions. Accounts of chemical research 50(2), 302–309 (2017) 28182403 10.1021/acs.accounts.6b00491

[26] Stärk H., Ganea O., Pattanaik L., Barzilay R., Jaakkola T.: Equibind: Geometric deep learning for drug binding structure prediction. In: International Conference on Machine Learning, pp. 20503–20521 (2022). PMLR

[27] DeLano W.L., : Pymol: An open-source molecular graphics tool. CCP4 Newsl. Protein Crystallogr 40(1), 82–92 (2002)

[28] Robin X., Studer G., Durairaj J., Eberhardt J., Schwede T., Walters W.P.: Assessment of protein–ligand complexes in casp15. Proteins: Structure, Function, and Bioinformatics 91(12), 1811–1821 (2023) 10.1002/prot.26601 37795762

[29] Bouysset C., Fiorucci S.: Prolif: a library to encode molecular interactions as fingerprints. Journal of cheminformatics 13(1), 72 (2021) 34563256 10.1186/s13321-021-00548-6 PMC8466659

[30] Mariani V., Biasini M., Barbato A., Schwede T.: lddt: a local superposition-free score for comparing protein structures and models using distance difference tests. Bioinformatics 29(21), 2722–2728 (2013) 23986568 10.1093/bioinformatics/btt473 PMC3799472

[31] Errington D., Schneider C., Bouysset C., Dreyer F.A.: Assessing interaction recovery of predicted protein-ligand poses. In: NeurIPS Workshop Foundation Models for Science: Progress, Opportunities, and Challenges (2024)

[32] Rubner Y., Tomasi C., Guibas L.J.: The earth mover’s distance as a metric for image retrieval. International journal of computer vision 40, 99–121 (2000)

[33] Virtanen P., Gommers R., Oliphant T.E., Haberland M., Reddy T., Cournapeau D., Burovski E., Peterson P., Weckesser W., Bright J., : Scipy 1.0: fundamental algorithms for scientific computing in python. Nature methods 17(3), 261–272 (2020) 32015543 10.1038/s41592-019-0686-2 PMC7056644

[34] Trott O., Olson A.J.: Autodock vina: improving the speed and accuracy of docking with a new scoring function, efficient optimization, and multithreading. Journal of computational chemistry 31(2), 455–461 (2010) 19499576 10.1002/jcc.21334 PMC3041641

[35] Krivák R., Hoksza D.: P2rank: machine learning based tool for rapid and accurate prediction of ligand binding sites from protein structure. Journal of cheminformatics 10, 1–12 (2018) 30109435 10.1186/s13321-018-0285-8 PMC6091426

[36] Passaro S., Corso G., Wohlwend J., Reveiz M., Thaler S., Somnath V.R., Getz N., Portnoi T., Roy J., Stark H., : Boltz-2: Towards accurate and efficient binding affinity prediction. BioRxiv, 2025–06 (2025)

[37] Lu W., Wu Q., Zhang J., Rao J., Li C., Zheng S.: Tankbind: Trigonometry-aware neural networks for drug-protein binding structure prediction. Advances in neural information processing systems 35, 7236–7249 (2022)

[38] Yim J., Stärk H., Corso G., Jing B., Barzilay R., Jaakkola T.S.: Diffusion models in protein structure and docking. Wiley Interdisciplinary Reviews: Computational Molecular Science 14(2), 1711 (2024)

[39] Zhang X., Zhang O., Shen C., Qu W., Chen S., Cao H., Kang Y., Wang Z., Wang E., Zhang J., : Efficient and accurate large library ligand docking with karmadock. Nature Computational Science 3(9), 789–804 (2023) 38177786 10.1038/s43588-023-00511-5

[40] Masters M., Mahmoud A., Lill M.: Fusiondock: Physics-informed diffusion model for molecular docking. In: ICML2023 CompBio Workshop (2023)

[41] Plainer M., Toth M., Dobers S., Stark H., Corso G., Marquet C., Barzilay R.: Diffdock-pocket: Diffusion for pocket-level docking with sidechain flexibility. NeurIPS 2023 Machine Learning in Structural Biology Workshop (2023)

[42] Guo H., Liu S., Mingdi H., Lou Y., Jing B.: Diffdock-site: A novel paradigm for enhanced protein-ligand predictions through binding site identification. In: NeurIPS 2023 Generative AI and Biology (GenBio) Workshop (2023)

[43] Pei Q., Gao K., Wu L., Zhu J., Xia Y., Xie S., Qin T., He K., Liu T.-Y., Yan R.: Fabind: Fast and accurate protein-ligand binding. Advances in Neural Information Processing Systems 36 (2024)

[44] Zhu J., Gu Z., Pei J., Lai L.: Diffbindfr: An se (3) equivariant network for flexible protein-ligand docking. Chemical Science (2024) 10.1039/d3sc06803j PMC11134415 38817560

[45] Cao D., Chen M., Zhang R., Wang Z., Huang M., Yu J., Jiang X., Fan Z., Zhang W., Zhou H., : Surfdock is a surface-informed diffusion generative model for reliable and accurate protein–ligand complex prediction. Nature Methods, 1–13 (2024) 39604569 10.1038/s41592-024-02516-y

[46] Huang Y., Zhang O., Wu L., Tan C., Lin H., Gao Z., Li S., Li S., : Re-dock: Towards flexible and realistic molecular docking with diffusion bridge. arXiv preprint arXiv:2402.11459 (2024)

[47] Miñán R., Sáenz J G., Molina A., : Geodirdock: Guiding docking along geodesic paths. In: ICLR 2024 Workshop on Generative and Experimental Perspectives for Biomolecular Design

[48] Bryant P., Kelkar A., Guljas A., Clementi C., Noé F.: Structure prediction of protein-ligand complexes from sequence information with umol. Nature Communications 15(1), 4536 (2024) 10.1038/s41467-024-48837-6 PMC11133481 38806453

[49] Stark H., Jing B., Barzilay R., Jaakkola T.: Harmonic self-conditioned flow matching for joint multi-ligand docking and binding site design. In: Forty-first International Conference on Machine Learning

[50] Morehead A., Cheng J.: Flowdock: Geometric flow matching for generative protein-ligand docking and affinity prediction. arXiv preprint arXiv:2412.10966 (2024) 10.1093/bioinformatics/btaf187 PMC12261468 40662794

[51] Corso G., Somnath V.R., Getz N., Barzilay R., Jaakkola T., Krause A.: Flexible docking via unbalanced flow matching. In: ICML’24 Workshop ML for Life and Material Science: From Theory to Industry Applications (2024)

[52] Qiao Z., Ding F., Dresselhaus T., Rosenfeld M.A., Han X., Howell O., Iyengar A., Opalenski S., Christensen A.S., Sirumalla S.K., : Neuralplexer3: Physio-realistic biomolecular complex structure prediction with flow models. arXiv preprint arXiv:2412.10743 (2024)

[53] Yu Y., Lu S., Gao Z., Zheng H., Ke G.: Do deep learning models really outperform traditional approaches in molecular docking? In: ICLR 2023-Machine Learning for Drug Discovery Workshop

[54] Jain A.N., Cleves A.E., Walters W.P.: Deep-learning based docking methods: Fair comparisons to conventional docking workflows. arXiv preprint arXiv:2412.02889 (2024)

[55] Sharon D.A., Huang Y., Oyewole M., Mustafa S.: How to go with the flow: an analysis of flow matching molecular docking performance with priors of varying information content. In: ICLR 2024 Workshop on Generative and Experimental Perspectives for Biomolecular Design (2024)

[56] Hu Q., Wang Z., Meng J., Li W., Guo J., Mu Y., Wang S., Zheng L., Wei Y.: Opendock: a pytorch-based open-source framework for protein–ligand docking and modelling. Bioinformatics 40(11), 628 (2024) 10.1093/bioinformatics/btae628 PMC11552628 39432683

[57] Morehead A., Giri N., Liu J., Neupane P., Cheng J.: Assessing the potential of deep learning for protein-ligand docking. Zenodo (2025). https://doi.org/10.5281/zenodo.16791095. 10.5281/zenodo.16791095 PMC12851923 41624973

[58] Akhtar M., Benjelloun O., Conforti C., Giner-Miguelez J., Jain N., Kuchnik M., Lhoest Q., Marcenac P., Maskey M., Mattson P., Oala L., Ruyssen P., Shinde R., Simperl E., Thomas G., Tykhonov S., Vanschoren J., Vogler S., Wu C.-J.: Croissant: A Metadata Format for ML-Ready Datasets (2024)

[59] Zhang Y., Skolnick J.: Scoring function for automated assessment of protein structure template quality. Proteins: Structure, Function, and Bioinformatics 57(4), 702–710 (2004) 10.1002/prot.20264 15476259

[60] Buttenschoen M., Morris G.M., Deane C.M.: PoseBusters: AI-based docking methods fail to generate physically valid poses or generalise to novel sequences. Zenodo (2023). https://doi.org/10.48550/arXiv.2308.05777. 10.48550/arXiv.2308.05777 PMC10901501 38425520

[61] Stärk H., Jing B., Barzilay R., Jaakkola T.: Harmonic self-conditioned flow matching for multi-ligand docking and binding site design. arXiv preprint arXiv:2310.05764 (2023)

